# Supplementary material for: Smooth Interpolating Curves with Local Control and Monotone Alternating Curvature
Source: Comput Graph Forum. 2022 Oct 6;41(5):25–38. doi: 10.1111/cgf.14600 (PMC9827861; doi:10.1111/cgf.14600)
Supplement: Supplementary file 1 — Supplement Material [file CGF-41-25-s001.zip › Local-Smooth-Interpolating-MonoCurvature/extern/clothoids/submodules/Utils/docs/api-cpp/program_listing_file_Utils_ThreadPool.hxx.html]

Program Listing for File ThreadPool.hxx — Utils v1.2

### Navigation

- index
- toc
- Utils »
- Program Listing for File ThreadPool.hxx

# Program Listing for File ThreadPool.hxx¶

↰ Return to documentation for file (`Utils/ThreadPool.hxx`)

```
/*--------------------------------------------------------------------------*\
 |                                                                          |
 |  Copyright (C) 2020                                                      |
 |                                                                          |
 |         , __                 , __                                        |
 |        /|/  \               /|/  \                                       |
 |         | __/ _   ,_         | __/ _   ,_                                |
 |         |   \|/  /  |  |   | |   \|/  /  |  |   |                        |
 |         |(__/|__/   |_/ \_/|/|(__/|__/   |_/ \_/|/                       |
 |                           /|                   /|                        |
 |                           \|                   \|                        |
 |                                                                          |
 |      Enrico Bertolazzi                                                   |
 |      Dipartimento di Ingegneria Industriale                              |
 |      Universita` degli Studi di Trento                                   |
 |      email: enrico.bertolazzi@unitn.it                                   |
 |                                                                          |
\*--------------------------------------------------------------------------*/


#pragma once

#ifndef DOXYGEN_SHOULD_SKIP_THIS
#ifndef UTILS_THREADPOOL_dot_HH
#define UTILS_THREADPOOL_dot_HH
#endif

#include <algorithm>
#include <utility>
#include <vector>
//#include <type_traits>

#include <thread>
#include <condition_variable>
#include <mutex>
#include <functional>

#ifdef UTILS_OS_LINUX
  #include <pthread.h>
#endif

#ifdef max
  #undef max
#endif
#ifdef min
  #undef min
#endif

namespace Utils {

  #ifndef DOXYGEN_SHOULD_SKIP_THIS
  using std::pair;
  using std::vector;
  using std::atomic;
  using std::mutex;
  using std::condition_variable;
  using std::unique_lock;
  using std::thread;
  using std::function;
  using std::move;
  using std::bind;
  using std::forward;
  using std::memory_order_relaxed;
  using std::memory_order_acquire;
  using std::memory_order_release;
  using std::max;
  using std::min;
  #endif

  /*\
   |   _____ _                        _
   |  |_   _| |__  _ __ ___  __ _  __| |___
   |    | | | '_ \| '__/ _ \/ _` |/ _` / __|
   |    | | | | | | | |  __/ (_| | (_| \__ \
   |    |_| |_| |_|_|  \___|\__,_|\__,_|___/
  \*/

  class SpinLock {
    // see https://geidav.wordpress.com/2016/03/23/test-and-set-spinlocks/
  private:
    atomic<bool> m_locked = {false};
  public:
    SpinLock() {}

    void
    wait() {
      while (m_locked.load(memory_order_relaxed) == true);
    }

    void
    lock() {
      do { wait(); } while (m_locked.exchange(true, memory_order_acquire) == true);
    }

    void
    unlock() {
      m_locked.store(false, memory_order_release);
    }
  };

  // - - - - - - - - - - - - - - - - - - - - - - - - - - - - - - - - - - - - -

  class WaitWorker {
  private:
    #ifdef UTILS_OS_WINDOWS
    atomic<int> n_worker;
    #else
    atomic<int> n_worker = {0};
    #endif
  public:
    #ifdef UTILS_OS_WINDOWS
    WaitWorker() { n_worker = 0; }
    #else
    WaitWorker() {}
    #endif

    void
    wait() {
      while (n_worker.load(memory_order_relaxed) != 0 );
    }

    void enter() { ++n_worker; }
    void leave() { --n_worker; }
  };

  // - - - - - - - - - - - - - - - - - - - - - - - - - - - - - - - - - - - - -

  template <typename DATA>
  class BinarySearch {
  private:
    typedef pair<thread::id,DATA*> DATA_TYPE;
    mutable vector<DATA_TYPE>      m_data;
    mutable SpinLock               m_spin_write;
    mutable WaitWorker             m_worker_read;

  public:

    BinarySearch() {
      m_data.clear();
      m_data.reserve(64);
    }

    ~BinarySearch() {
      m_spin_write.wait();
      for ( auto & a : m_data ) delete a.second;
      m_data.clear();
      m_spin_write.wait();
    }

    void
    clear() {
      m_spin_write.wait();
      for ( auto & a : m_data ) delete a.second;
      m_data.clear(); m_data.reserve(64);
      m_spin_write.wait();
    }

    DATA *
    search( thread::id const & id, bool & ok ) const {
      m_spin_write.wait(); // wait writing finished
      m_worker_read.enter();
      ok = true;

      size_t U = m_data.size();

      if ( U == 0 ) {
        m_worker_read.leave();
        m_spin_write.lock();
        m_worker_read.wait(); // wait all read finished
        ok = false;
        U  = m_data.size();
        m_data.resize(1);
        DATA_TYPE & dL = m_data[0];
        dL.first = id;
        DATA * res = dL.second = new DATA();
        m_spin_write.unlock();
        return res;
      }

      size_t L = 0;
      while ( U-L > 1 ) {
        size_t pos = (L+U)>>1;
        thread::id const & id_pos = m_data[pos].first;
        if ( id_pos < id ) L = pos; else U = pos;
      }
      DATA_TYPE & dL = m_data[L];
      if ( dL.first == id ) { m_worker_read.leave(); return dL.second; }
      DATA_TYPE & dU = m_data[U];
      if ( dU.first == id ) { m_worker_read.leave(); return dU.second; }
      m_worker_read.leave();

      // not found must insert
      m_spin_write.lock();
      m_worker_read.wait(); // wait all read finished
      ok = false;
      if ( dL.first < id ) ++L;
      U = m_data.size();
      m_data.resize(U+1);
      while ( U > L ) {
        --U;
        m_data[U+1].first  = m_data[U].first;
        m_data[U+1].second = m_data[U].second;
      }
      DATA_TYPE & dL1 = m_data[L];
      dL1.first = id;
      DATA * res = dL1.second = new DATA();
      m_spin_write.unlock();
      return res;
    }

  };

  // - - - - - - - - - - - - - - - - - - - - - - - - - - - - - - - - - - - - -

  class SpinLock_barrier {
  private:
    atomic<unsigned> m_count;
    atomic<unsigned> m_generation;
    unsigned         m_count_reset_value;
  public:
    SpinLock_barrier( SpinLock_barrier const & ) = delete;
    SpinLock_barrier& operator=( SpinLock_barrier const & ) = delete;

    explicit
    SpinLock_barrier()
    : m_generation(0)
    {}

    void
    setup( unsigned count ) {
      m_count_reset_value = m_count = count ;
    }

    void
    count_down() {
      unsigned gen = m_generation.load();
      if ( --m_count == 0 ) {
        if ( m_generation.compare_exchange_weak(gen, gen + 1) )
          m_count = m_count_reset_value;
        return;
      }
    }

    void
    wait() {
      unsigned gen = m_generation.load();
      while ((gen == m_generation) && (m_count != 0))
        std::this_thread::yield();
    }

    void
    count_down_and_wait() {
      unsigned gen = m_generation.load();
      if ( --m_count == 0 ) {
        if ( m_generation.compare_exchange_weak(gen, gen + 1) )
          m_count = m_count_reset_value;
        return;
      }
      while ((gen == m_generation) && (m_count != 0))
        std::this_thread::yield();
    }
  };

  // - - - - - - - - - - - - - - - - - - - - - - - - - - - - - - - - - - - - -

  class Barrier {
    int                m_to_be_done;
    int                m_usedThread;
    mutex              m_mtx;
    condition_variable m_cond;
  public:
    Barrier() : m_to_be_done(0) {}

    void
    setup( int nthreads )
    { m_usedThread = m_to_be_done = nthreads ; }

    void
    count_down() {
      unique_lock<mutex> lck(m_mtx);
      if ( --m_to_be_done <= 0 ) m_cond.notify_all() ; // wake up all tread
    }

    void
    wait() {
      unique_lock<mutex> lck(m_mtx);
      m_cond.wait(lck);
    }

    void
    count_down_and_wait() {
      unique_lock<mutex> lck(m_mtx);
      if ( --m_to_be_done <= 0 ) {
        m_cond.notify_all() ; // wake up all tread
        m_to_be_done = m_usedThread ;
      } else {
        m_cond.wait(lck);
      }
    }
  };

  // - - - - - - - - - - - - - - - - - - - - - - - - - - - - - - - - - - - - -

  class SimpleSemaphore {
  private:
    bool               m_go;
    mutex              m_mutex;
    condition_variable m_cv;
  public:
    SimpleSemaphore() noexcept : m_go(true) {}

    void
    green() noexcept {
      { unique_lock<mutex> lock(m_mutex); m_go = true; }
      m_cv.notify_one();
    }

    void
    red() noexcept {
      { unique_lock<mutex> lock(m_mutex); m_go = false; }
      m_cv.notify_one();
    }

    void
    wait() noexcept {
      unique_lock<mutex> lock(m_mutex);
      m_cv.wait(lock, [this]()->bool { return this->m_go; });
    }

  };

  /*\
   |  __        __         _
   |  \ \      / /__  _ __| | _____ _ __
   |   \ \ /\ / / _ \| '__| |/ / _ \ '__|
   |    \ V  V / (_) | |  |   <  __/ |
   |     \_/\_/ \___/|_|  |_|\_\___|_|
  \*/

  class Worker {
    friend class ThreadPool;

    bool             m_active;
    SimpleSemaphore  m_is_running;
    SimpleSemaphore  m_job_done;
    thread           m_running_thread;
    function<void()> m_job;

    //disable copy
    Worker( Worker const & ) = delete;
    Worker& operator = ( Worker const & ) = delete;

    void
    loop() {
      while ( m_active ) {
        m_is_running.wait();
        if ( m_active ) m_job();
        m_is_running.red();
        m_job_done.green();
      }
    }

  public:

    Worker() : m_active(false) { start(); }
    ~Worker() { stop(); }

    Worker( Worker && rhs ) {
      m_active         = rhs.m_active;
      m_job            = rhs.m_job;
      m_running_thread = move(rhs.m_running_thread);
    }

    void
    start() {
      if ( !m_active ) {
        m_active = true;
        m_is_running.red();
        m_job_done.green();
        m_running_thread = thread( [this] () -> void { this->loop(); } );
      }
    }

    void
    stop() {
      if ( m_active ) {
        m_active = false;        // deactivate computation
        m_is_running.green();    // for exiting from the loop
        m_running_thread.join(); // wait thread for exiting
      }
    }

    void wait() { m_job_done.wait(); }

    template < class Func, class... Args >
    void
    run( Func && func, Args && ... args ) {
      //launch( bind(forward<Func>(func), forward<Args>(args)...) );
      m_job_done.wait(); // se gia occupato in task aspetta
      m_job = bind(forward<Func>(func),forward<Args>(args)...);
      m_job_done.red();
      m_is_running.green(); // activate computation
    }

  };

  /*\
   |   _____ _                        _ ____             _
   |  |_   _| |__  _ __ ___  __ _  __| |  _ \ ___   ___ | |
   |    | | | '_ \| '__/ _ \/ _` |/ _` | |_) / _ \ / _ \| |
   |    | | | | | | | |  __/ (_| | (_| |  __/ (_) | (_) | |
   |    |_| |_| |_|_|  \___|\__,_|\__,_|_|   \___/ \___/|_|
  \*/

  class ThreadPool {
    // need to keep track of threads so we can join them
    vector<Worker> m_workers;

    //disable copy
    ThreadPool() = delete;
    ThreadPool( ThreadPool const & ) = delete;
    ThreadPool & operator = ( ThreadPool const & ) = delete;

    #if defined(UTILS_OS_WINDOWS)
    void
    setup() {
      for ( auto & w: m_workers ) w.start();
    }
    #elif defined(UTILS_OS_LINUX)
    void
    setup() {
      sched_param sch;
      int         policy;
      for ( auto & w: m_workers ) {
        w.start();
        thread & t = w.m_running_thread;
        pthread_getschedparam( t.native_handle(), &policy, &sch );
        sch.sched_priority = sched_get_priority_max( SCHED_RR );
        pthread_setschedparam( t.native_handle(), SCHED_RR, &sch );
      }
    }
    #else
    void
    setup() {
      for ( auto & w: m_workers ) w.start();
    }
    #endif

  public:

    ThreadPool(
      unsigned nthread = max(
        unsigned(1),
        unsigned(thread::hardware_concurrency()-1)
      )
    ) {
      m_workers.resize( size_t( nthread ) );
      setup();
    }

    template <typename Func, typename... Args>
    void
    run( unsigned nt, Func && func, Args && ... args ) {
      m_workers[size_t(nt)].run( func, args...);
    }

    void wait_all()  { for ( auto && w : m_workers ) w.wait(); }
    void start_all() { for ( auto && w : m_workers ) w.start(); }
    void stop_all()  { for ( auto && w : m_workers ) w.stop(); }

    unsigned size() const { return unsigned(m_workers.size()); }

    thread::id
    get_id( unsigned i ) const
    { return m_workers[size_t(i)].m_running_thread.get_id(); }

    thread const &
    get_thread( unsigned i ) const
    { return m_workers[size_t(i)].m_running_thread; }

    thread &
    get_thread( unsigned i )
    { return m_workers[size_t(i)].m_running_thread; }

    void
    resize( unsigned numThreads ) {
      wait_all();
      stop_all();
      m_workers.resize( size_t(numThreads) );
      setup();
    }

  };

}

#endif
```

### Quick search

### Table of Contents

- rang - Colors for your Terminal
- Zstr
- C++ API

«
hide menu

menu
sidebar
»

### Navigation

- index
- toc
- Utils »
- Program Listing for File ThreadPool.hxx

© Copyright 2021, Enrico Bertolazzi.
Created using Sphinx 4.2.0.
